# Multiple infection of cells changes the dynamics of basic viral evolutionary processes

**DOI:** 10.1002/evl3.95

**Published:** 2018-12-31

**Authors:** Dominik Wodarz, David N. Levy, Natalia L. Komarova

**Affiliations:** ^1^ Department of Ecology and Evolutionary Biology, 321 Steinhaus Hall University of California Irvine CA 92697; ^2^ Department of Mathematics, Rowland Hall University of California Irvine CA 92697; ^3^ Department of Basic Science, 921 Schwartz Building New York University College of Dentistry New York NY 10010

**Keywords:** Evolution, evolutionary dynamics, fixation probability, mathematical models, multiple infection, virus dynamics

## Abstract

The infection of cells by multiple copies of a given virus can impact viral evolution in a variety of ways, yet some of the most basic evolutionary dynamics remain underexplored. Using computational models, we investigate how infection multiplicity affects the fixation probability of mutants, the rate of mutant generation, and the timing of mutant invasion. An important insight from these models is that for neutral and disadvantageous phenotypes, rare mutants initially enjoy a fitness advantage in the presence of multiple infection of cells. This arises because multiple infection allows the rare mutant to enter more target cells and to spread faster, while it does not accelerate the spread of the resident wild‐type virus. The rare mutant population can increase by entry into both uninfected and wild‐type‐infected cells, while the established wild‐type population can initially only grow through entry into uninfected cells. Following this initial advantageous phase, the dynamics are governed by drift or negative selection, respectively, and a higher multiplicity reduces the chances that mutants fix in the population. Hence, while increased infection multiplicity promotes the presence of neutral and disadvantageous mutants in the short‐term, it makes it less likely in the longer term. We show how these theoretical insights can be useful for the interpretation of experimental data on virus evolution at low and high multiplicities. The dynamics explored here provide a basis for the investigation of more complex viral evolutionary processes, including recombination, reassortment, as well as complementary/inhibitory interactions.

**Impact summary**

The infection of cells by multiple copies of a given virus can impact virus evolution in a variety of ways, for example, through recombination and reassortment, or through intracellular interactions among the viruses in a cell, such as complementation or interference. Surprisingly, multiple infection of cells can also influence some of the most basic evolutionary processes, which has not been studied in detail so far. Here, we use computational models to explore how infection multiplicity affects the fixation probability of mutants, the rate of mutant generation, and the timing of mutant invasion. This is investigated for neutral, disadvantageous, and advantageous mutants. Among the results, we note surprising growth dynamics for neutral and disadvantageous mutants when rare: Starting from a single mutant‐infected cell, an initial growth phase is observed, which is more characteristic of an advantageous mutant and is not observed in the absence of multiple infection. Therefore, in the short term, multiple infection increases the chances that neutral or disadvantageous mutants are present. Following this initial growth phase, however, the mutant dynamics enter a second phase that is driven by neutral drift or negative selection, respectively, which determines the long‐term fixation probability of the mutant. Contrary to the short‐term dynamics, the probability of mutant fixation, and thus existence, is lower in the presence compared to the absence of multiple infection, and declines with infection multiplicity. Hence, while infection multiplicity promotes mutant existence in the short‐term, it makes it less likely in the longer term. Understanding these dynamics is useful for the interpretation of experimental data and forms the basis for the investigation of more complex viral evolutionary processes.

RNA viruses are characterized by a large amount of genetic diversity that allows rapid adaptation to environmental challenges, due to relatively high mutation rates, large population sizes, and rapid replication (Domingo et al. 1996; Domingo and Holland 1997). The evolutionary dynamics of RNA viruses have been extensively studied in a variety of contexts (Elena and Lenski 2003; Moya et al. 2004; Lauring and Andino 2010). Much of this work has viewed the virus genome as a solitary entity, where a specific gene in a given virus maps directly to its phenotype. It has, however, been demonstrated experimentally that genetically diverse viruses of the same species frequently co‐habit a single cell, resulting in a variety of positive and negative interactions (Sakai 1955; Griffing 1967; Moore et al. 1997; Wolf et al. 1998; Wolf 2000; Frank 2007; Bijma 2014) that can determine the response to selection and the level of genetic variation in the population (Mutic and Wolf 2007; Ojosnegros et al. 2011; Peeters et al. 2012; Bijma 2014). Viral complementation has been observed in several cases, leading to the persistence of inferior mutants (Froissart et al. 2004; Garcia‐Arriaza et al. 2004, 2006; Gelderblom et al. 2008). Negative interactions range from straightforward competitive interactions between viruses in a cell to the inhibition of the viral replicative potential (de la Torre and Holland 1990; Chumakov et al. 1991; Ojosnegros et al. 2011). Furthermore, different viruses coinfecting the same cell can exchange genetic information by recombination for retroviruses such as HIV, and by reassortment for segmented viruses (e.g., influenza virus).

The effect of infection multiplicity (virus copies per cell) on evolutionary outcome has been examined experimentally in a variety of setting (de la Torre and Holland 1990; Chumakov et al. 1991; Turner and Chao 1999, 2003; Garcia‐Arriaza et al. 2004, 2006; Dennehy et al. 2013; Donahue et al. 2013). For example, in experiments with RNA phage φ6, defectors evolved at high infection multiplicities that lowered the fitness of the phage population, which was not observed at low multiplicities (Turner and Chao 1999, 2003). In a different study, viral diversity was found to be lower at high infection multiplicities, arguing that viral segmentation might have evolved for reasons other than the benefits of sex (Dennehy et al. 2013). In the context of HIV‐1, multiple infection has been shown to influence the latent state of integrated viruses (Donahue et al. 2013). Overall, such work has shown that the effect of multiple infection on evolution is multifactorial and complex.

From a mathematical point of view, aspects of viral dynamics have been investigated in the context of multiple infection (Dixit and Perelson 2004, 2005; Wodarz and Levy 2011; Cummings et al. 2012; Komarova et al. 2012, 2013; Asatryan et al. 2015), including studies of competition dynamics and evolutionary processes (May and Nowak 1994; Nowak and May 1994; vanBaalen and Sabelis 1995; Alizon and van Baalen 2008; Lipsitch et al. 2009; Alizon et al. 2013; Phan and Wodarz 2015; Leeks et al. 2018), especially with a focus on recombination (Bretscher et al. 2004; Althaus and Bonhoeffer 2005; Fraser 2005; Kouyos et al. 2006; Vijay et al. 2008). The effect of infection multiplicity on more basic evolutionary measures, such as the fixation probability of mutants, the time until mutant generation, and time until mutant invasion or fixation, has, however, so far not been investigated in detail. The present study aims to fill this gap. A solid understanding of the effect of multiplicity on basic evolutionary processes forms the underpinning for exploring more complicated scenarios in future work, including investigations into the effect of intracellular interactions among different viruses within the same cell on evolutionary outcome.

## The Computational Modeling Framework

We study the evolutionary dynamics with a stochastic agent‐based model because this allows for a natural formulation of the multiple infection process (Phan and Wodarz 2015). The model consists of *N* spots, which can be either empty, contain an uninfected cell, or contain an infected cell. Every time step, the system is randomly sampled *N* times, and the chosen spots are updated according to specific rules (see Fig. S1 in Supporting Information for schematic representation). If the chosen spot is empty, there is a probability *L* to produce an uninfected cell. If the sampled spot contains an uninfected cell, it can die with a probability *D*. If the sampled spot contains an infected cell, two events can happen. The cell can die with a probability *A*, and it can initiate an infection event with a probability *B*. If an infection event is initiated, a target spot is chosen randomly from the whole system. If that spot contains a susceptible cell, the infection event occurs, otherwise it is aborted. If the susceptible cell is an uninfected cell, it becomes infected with one virus. If the cell is already infected, its multiplicity is increased by one. The probability of an infected cell dying, as well as the probability of transmitting a virus to another cell is assumed to be independent of infection multiplicity (this will apply to all models unless otherwise stated, and different assumptions are explored in the Supporting Information and summarized below). The model assumes perfect mixing of viruses and cells. The average behavior of this system can be described by ordinary differential equations (ODEs, see Section 2, Supporting Information). If the basic reproductive ratio of the virus (Nowak and May 2000) is greater than one, the dynamics converge toward a stable equilibrium that is also defined in the Supporting Information. In the stochastic simulations, the populations fluctuate around this equilibrium.

When a mutant virus is considered, there are two virus strains in the system that need to be tracked. The agent‐based model follows cell populations that contain *i* copies of the wild‐type virus, and *j* copies of the mutant virus. If a coinfected cell is chosen for infection, the virus strain to be transmitted is chosen randomly based on the fraction of the virus in the cell. Thus, the wild‐type virus is chosen with a probability given by *i*/(*i* + *j*), and the mutant virus is chosen with probability *j*/(*i + j*) (Phan and Wodarz 2015). These assumptions can also be expressed in terms of ODEs, as shown in the Supporting Information (Section 2).

## Varying the Infection Multiplicity

The goal of this work is to compare the evolutionary dynamics in settings where the multiplicity of infected cells is varied. This is achieved by increasing the infection probability *B*, because higher infection probabilities correlate with larger infection multiplicities at equilibrium, as shown in Section 3 of the Supporting Information.

## Evolutionary Dynamics of Neutral Mutants

We first consider the evolutionary spread of neutral mutants, that is, the model parameters of the wild‐type and mutant are identical. Different evolutionary endpoints will be considered in turn.

### MUTANT FIXATION PROBABILITY

**Figure 1:**
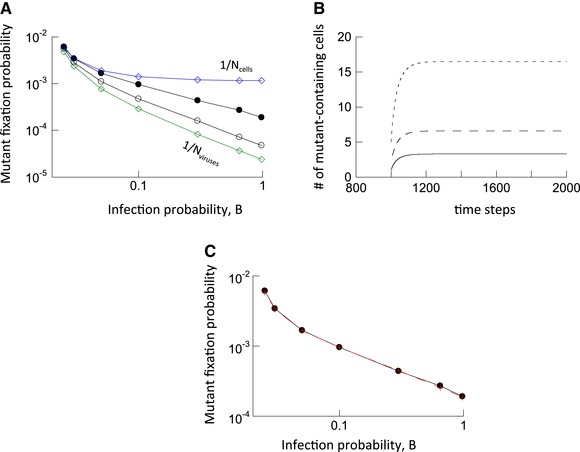
Evolutionary dynamics of neutral mutants. (A) Fixation probability as a function of the infection probability, B. Two theoretical bounds are shown by diamonds. The blue line with diamonds shows the fixation probability in the absence of multiple infection, given by 1/*N*_cells_, where *N*_cells_ is the equilibrium number of infected cells before mutant introduction. The green line with diamonds shows 1/*N*_viruses_, where *N*_viruses_ is the equilibrium number of viruses across all cells before mutant introduction, and was hypothesized to be the theoretically expected fixation probability in the presence of multiple infection. The circles show results of two types of computer simulations. The black closed circles show the fixation probabilities in the computer simulation when one cell infected with one mutant virus is introduced into the system, where the wild‐type virus population has equilibrated. The black open circles show the fixation probabilities in the computer simulation when one mutant virus is randomly placed into any of the available cells in the system where the wild‐type virus population has equilibrated. Base parameters were: A = 0.02, L = 1, D = 0.01, *N* = 900. The number of simulation runs varied for different parameters due to different speeds of the computer simulation. For the black circles, the numbers of runs for increasing values of B were: 14,154,839; 15,577,853; 10,415,129; 18,733,054; 8,590,117; 5,814,742; and 4,518,280. For open circles, the numbers of runs were: 29,237,576; 33,491,598; 24,902,642; 33,231,461; 28,297,798; 22,471,381; and 46,938,021. The trends described in the text are statistically significant, according to the *Z* test for two population proportions (very low *P*‐values, not shown). (B) Average dynamics of neutral mutants following introduction into a system at equilibrium, given by ODE model (2) in the Supporting Information. The different lines depict simulations that start from different initial conditions. We observe first a phase of mutant spread, followed by convergence to a neutrally stable equilibrium. Parameters were: *β* = 0.025, *a* = 0.02, *λ* = 1, *d* = 0.01, and *k* = 900. (C) Successful theoretical prediction of the observed mutant fixation probability. The black circles show the observed fixation probabilities, which are the same as in panel A. The red crosses plot the values of *N*_neut_/*N*_viruses_, which accurately predict the observed fixation probabilities, as explained in the text.

We initialized the agent‐based simulation by placing one cell with a single copy of the mutant virus (and no wild‐type virus) into a population where the wild‐type virus was present at equilibrium levels. The computer simulation was run repeatedly, and the fraction of simulations were determined that resulted in the fixation of the mutant. This is defined by the presence of the mutant virus, while the wild‐type virus has gone extinct; realizations of the simulation in which both populations went extinct were not observed, and the simulation was set up to not count such events should they occur. The mutant fixation probability was determined for increasing infections rates, which correlate with higher infection multiplicities (Fig. S3). Systems with and without multiple infection were compared. For simulations without multiple infection, infection events were aborted if the target cell already contained a virus. In the absence of multiple infection, the fixation probability of a neutral mutant is given by 1/*N*_cells_, where *N*_cells_ denotes the number of wild‐type‐infected cells at equilibrium before mutant introduction (Nei 1975; Hartl and Clark 1997; Ewens 2004; blue line, Fig. 1A). This was verified by numerical simulations (not shown). The simulation results in the presence of multiple infection are shown in 1A (black line, solid circles). For relatively low infection multiplicities (low infection probability, *B*), the observed fixation probability converges to the values in the absence of multiple infection, which is expected. The fixation probability, however, declines with increasing multiplicity, below the levels seen in the absence of multiple infection. Using the intuition from the theory of neutral evolution (Nei 1975; Hartl and Clark 1997; Ewens 2004), in the presence of multiple infection, the fixation probability should be given by 1/*N*_viruses_, where *N*_viruses_ is the total number of viruses across all cells in the system; this is shown by the green line in Figure 1A. The observed fixation probability of the neutral mutant (black closed circles, Fig. 1A), however, is significantly higher than this.

This discrepancy arises because there are two phases in the virus dynamics. The average mutant dynamics are shown in Figure 1B, based on simulations of ODE model (2) in the Supporting Information. We observe that the population of mutant infected cells (which includes all cells that contain at least one mutant) initially grows, as if it were advantageous. This is followed by convergence toward a neutrally stable equilibrium (denoted by *N*_neut_, which depends on the initial mutant population size, Fig. 1B). The initial growth phase, and hence, the initial advantage of the mutant, derives from the fact that in addition to uninfected cells, wild‐type‐infected cells also provide a target for new mutant infections. In contrast, new wild‐type‐infected cells can initially only be generated by viral entry into uninfected cells, since superinfection of wild‐type‐infected cells by more wild‐type‐virus does not result in the spread of the wild‐type virus population. As the mutant spreads, this advantage diminishes and the dynamics enter the long‐term neutral phase. This is because the mutant viruses become distributed among cells also containing wild‐type virus and the initial asymmetry in growth dynamics vanishes. The initial advantageous phase of the dynamics accounts for the observed fixation probability that is higher than expected from the straightforward application of the neutral evolution argument. In fact, the number of mutant viruses (across all cells) at this Neutral equilibrium, *N*_neut_, predicts the fixation probability, which is given by *N*_neut_/*N*_viruses_, where *N*_viruses_ is the total number of viruses before introduction of the mutant. This is shown in Figure 1C, where simulation results (black) are compared to the value of *N*_neut_/*N*_viruses_ (red). For this calculation, *N*_neut_ is determined by numerical integration of the ODEs.

In Figure 1A, the line with open circles depicts the results of additional simulations, which started from different initial conditions. Instead of introducing one cell that contains a single mutant virus, the mutant was placed into a randomly chosen (possibly infected) cell after the wild‐type population had equilibrated. The fraction of runs in which mutants reached fixation was recorded. This corresponds to a scenario where the mutant was generated from the wild‐type virus by mutational processes, and the fate of this mutant was followed for each realization of the simulation. Because mutant placement into a cell was probabilistic, in each simulation, the mutant virus was introduced into a different configuration, co‐resident with different numbers of wild‐type viruses within the cell. As seen in Figure 1A, the decline of the observed fixation probability of the neutral mutant with higher infection multiplicities is more pronounced in this case, and the fixation probability is closer to the value of 1/*N*_viruses_, but still higher. This makes intuitive sense, because the initial “advantageous” phase of the mutant dynamics is now less pronounced, due to intracellular competition of the first mutant virus with the wild‐type.

In these models, it was assumed that the multiplicity of infection is only limited by kinetic parameters, such as the viral replication rate and the death rate of infected cells. With several viruses, however, it has been documented that infection multiplicity might be artificially limited by viruses (Turner et al. 1999; Levy et al. 2004), for example, through receptor downmodulation (Levesque et al. 2004). Section 6 of the Supporting Information shows that basic results remain robust in this setting, and we discuss the ways in which evolutionary outcomes might differ. Another possible deviation from model assumptions is the observation that in certain settings, the fraction of multiply infected cells is larger than expected by chance (Turner et al. 1999; Law et al. 2016). The reasons are not fully understood. It can arise from differential susceptibility of cells to infection (Law et al. 2016), in which case our results would remain robust.

### TIME TO APPEARANCE OF FIRST MUTANT

**Figure 2:**
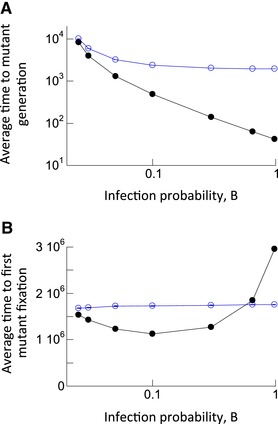
(A) Average time to generation of first mutant in the agent‐based model with mutations. Black closed circles denote the simulation results in the presence of multiple infection, and blue open circles denote simulation results in the absence of multiple infection. Standard errors are shown, but are relatively small and hard to see. The number of simulation runs for increasing values of B for black circles are: 166,137; 403,110; 906,346; 8,000,789; 8,992,529; 15,656,759; and 19,553,451. For blue circles: 2,159,214; 4,376,870; 5,980,191; 10,651,321; 11,229,080; 10,103,400; and 22,652,139. (B) Average time until the number of mutant‐infected cells reached 90% of the whole infected cell population in the model with mutation and back‐mutation (neutral mutants). The black closed circles show simulation results in the presence of multiple infection, the blue open circles show results without multiple infection. The simulation was started with the wild‐type virus population at equilibrium. Parameters were chosen as follows: B = 0.025, A = 0.02, L = 1, D = 0.01, μ = 3 × 10^−5^, *N* = 900. Standard errors are shown, which, however, are very small and hard to see. For increasing values of B, the number of simulations for the black circles was: 27,629; 34,858; 29,688; 42,050; 30,574; 39,744; and 20,570. For blue circles: 34,419; 39,953; 29,128; 38,395; 34,234; 72,679; and 64,963. Trends of how multiple infection affects the plotted measures are statistically significant according to the two‐sample *t*‐test (very low *P*‐values, not shown).

Another important evolutionary observable is the rate with which mutants are generated. This is explored here by quantifying the time it takes until the first mutant has been generated. To do this, we used a model that included mutational processes. A mutant is assumed to differ from the wild‐type by a single point mutation. When a wild‐type virus was chosen for transmission to a new cell, it was assumed that a point mutation occurred with a rate *p*_mut_. Biologically, this can correspond to point mutations that occur upon production of the offspring virus, or that occur during the subsequent infection event, such as in retroviruses (see Section 1, Supporting Information). We determined the time at which a newly produced mutant successfully entered a target cell for the first time. For practical purposes, we chose a relatively high point mutation rate of *p*_mut_ = 3.5 × 10^−5^ per base pair per generation, which is the mutation rate characteristic of HIV (Mansky and Temin 1995). The dependence on infection multiplicity was explored in the same way as described above, by varying the infection probability. We found that for all infection rates, the time to first mutant generation is always faster in the presence compared to the absence of multiple infection (Fig. 2A, compare black and blue line). Further, a higher infection multiplicity (infection rate) reduced the time at which the first mutant was generated (Fig. 2A). This makes intuitive sense. A higher infection rate/multiplicity corresponds to a larger number of successful replication events, during which mutations can occur.

### TIME TO MUTANT FIXATION

The above results indicate the existence of a trade‐off with respect to the effect of infection multiplicity. A higher infection multiplicity results in the more frequent generation of mutants. At the same time, however, it also leads to a lower probability of such mutants to fixate. The current section explores this tradeoff by using the model version with mutational processes and determining the time it takes for the mutant population to invade. In addition to wild‐type giving rise to mutant viruses, however, we also need to account for back mutations, since this counteracts the mutant expansion dynamics. In these simulations, the mutants are repeatedly generated (and eliminated at the same rate) and drift stochastically. Because of the occurrence of back‐mutations, mutant fixation is not an absorbing state. To capture the effect of the trade‐off between increased mutant production and reduced invasion potential, we therefore recorded the time until the mutant fixes for the first time (we refer to this event as “mutant invasion”). The results are shown by black circles in Figure 2B as a function of infection multiplicity. The corresponding results for simulations without multiple infection are shown in the blue line (Fig. 2B). We find that multiplicity influences the time to mutant invasion in a non‐monotonous way. For low viral infection rates (and hence low infection multiplicities), an increase in infection rate and multiplicity results in a reduced time to mutant invasion, which is below the time observed without multiple infection. As the infection rate and multiplicity are increased further, however, the time to mutant invasion becomes longer and rises above that observed in the absence of multiple infection (Figure 2B). Therefore, for moderate infection multiplicities, multiple infection speeds up mutant invasion. For higher infection multiplicities, multiple infection slows down mutant invasion. To show the importance of back mutations in this process, we compared results to those obtained in a model without back mutations in Supporting Information Section 5, where the rise in the mutant fixation time at high multiplicities was less pronounced.

## Disadvantageous Mutants

**Figure 3:**
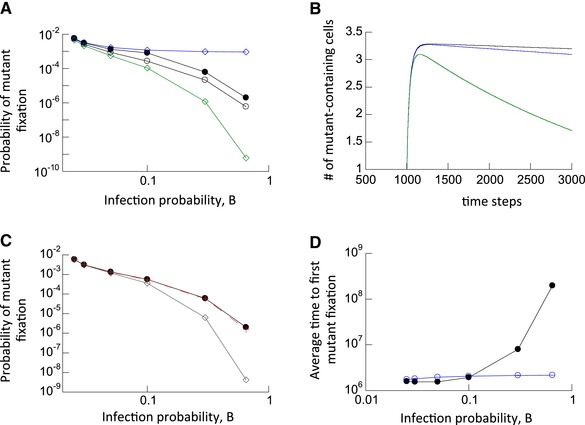
Evolutionary dynamics of disadvantageous mutants. (A) Fixation probability as a function of the infection probability, B. Two theoretical bounds are shown by diamonds. The blue line with diamonds shows the fixation probability in the absence of multiple infection as given by formula (1) derived from the Moran process. The green line shows the fixation probability according to formula (2) derived from the Moran process. The black closed circles show the fixation probabilities observed in the agent based simulation when one cell infected with one mutant virus is introduced into the system at equilibrium. The black open circles show the fixation probabilities observed in the agent based simulation when one mutant virus is randomly placed into any of the available cells in system at equilibrium. Base parameters were: B_1_ = 0.025, B_2_ = rB_1_, A = 0.02, L = 1, D = 0.01, μ = 3 × 10^−5^, *r* = 0.9995. The number of simulation runs for increasing values of B for the black closed circles were: 101,317,577; 112,619,340; 77,957,298; 37,907,585; 72,473,679; and 47,395,056. For open black circles: 196,598,760; 225,295,595; 168,947,826; 227,879,849; 199,753,392; and 277,735,577. Trends described in the text are statistically significant, according to the *Z* test for two population proportions (very low p values, not shown). (B) Average dynamics of disadvantageous mutants following introduction into a system at equilibrium, given by ODE model (2) in the Supporting Information. We observe first a phase of mutant spread, followed by a decline toward extinction. Different lines depict different levels of mutant disadvantage. A larger disadvantage leads to a less pronounced initial spread phase, followed by a faster decline. Parameters were: β_1_ = 0.025, β_2_ = r β_1_ a = 0.02, λ = 1, d = 0.01, k = 900. The relative mutant fitness values were (from top to bottom) *r* = 0.9995, *r* = 0.999, and *r* = 0.99. (C) Predicting the fixation probability of disadvantageous mutants. The black closed circles depict the same mutant fixation probabilities as in panel (A), observed in agent based simulations that started with one cell containing one mutant virus with *r* = 0.9995. The line with grey diamonds depicts the value of formula (3), assuming an initial mutant virus population size of *N*_neut_, and the relative mutant fitness disadvantage *r* = 0.9995. This fails to accurately predict the observed fixation probability. The red line with crosses depicts the same formula (3), but using the composite mutant fitness value *r*’, defined in formula (4) in the text. This accurately predicts the observed fixation probability. (D) Average time until the number of infected cells containing the disadvantageous mutant reached 90% of the whole infected cell population, given by the agent‐based model with mutations and back‐mutations (black circles). The blue line depicts the same measure in the absence of multiple infection, determined by simulations of the agent‐based model. Parameters were: B_1_ = 0.025, B_2_ = rB_1_, A = 0.02, L = 1, D = 0.01, μ = 3 × 10^−5^, *N* = 900, *r* = 0.9995. Standard errors are shown but are relatively small and hard to see. The trends described in the text are statistically significant, according to the two‐sample *t*‐test. The number of simulation results for increasing values of B for the black line was: 323,339; 307,142; 281,610; 234,979; 46,647; and 1338. For the blue line: 294,547; 262,278; 224,520; 227,618; 201,695; and 168677.

Next, we studied the evolutionary dynamics of slightly disadvantageous (0.05% fitness cost) mutants. The rules of the model are identical to those assumed for neutral mutants. In addition, once a virus was picked to infect a target cell, we assumed that this process failed with a probability 0.05% if this virus was a mutant, while it always succeeded if the selected virus was wild‐type. In the absence of multiple infection, we numerically confirmed (not shown) that when one mutant‐infected cell is introduced into a wild‐type virus population at equilibrium, the fixation probability of the mutant is given by

(1) $$\frac{1-(1/r)}{1-1/r^{N_{\mathrm{cells}}}},$$

which is a formula derived from the Moran Process (Komarova et al. 2003). Here, *r* expresses the disadvantage of the mutant relative to the wild‐type, and *N*_cells_ denotes the number of wild‐type infected cells at equilibrium before the mutant is introduced (see blue line, Fig. 3A). In the context of multiple infection, the number of viruses rather than the number of cells should be the relevant population size, and hence, by extension, the equivalent fixation probability would be given by

(2) $$\frac{1-(1/r)}{1-1/r^{N_{\mathrm{viruses}}}},$$

where *N*_viruses_ denotes the number of viruses across all infected cells (for reference, this is plotted by the green line in Fig. 3A).

First, the simulations were started with one cell containing a single mutant virus being placed into a wild‐type virus population at equilibrium (black closed circles, Fig. 3A). Similar trends are observed compared to neutral mutants. The fixation probability of the disadvantageous mutant is found to be lower in the presence compared to the absence of multiple infection (Fig. 3A, black closed circles and blue diamonds), and decreases with higher infection multiplicities. This decrease of the fixation probability with higher infection multiplicity is more pronounced than for neutral mutants. Nevertheless, the mutant fixation probability observed in the simulations is significantly higher than the one predicted by formula (2) (green line, Fig. 3A). One reason for the higher fixation probability is the same as for neutral mutants. Despite its replicative disadvantage, the mutant initially enjoys an advantage over the wild‐type virus, because in addition to uninfected cells, it can also spread by entering wild‐type‐infected cells. Using the ODE model (2) in the Supporting Information, this is shown in Figure 3B. The mutant cell population first rises. This is followed by a decline phase toward extinction, due to the assumed replicative disadvantage. The peak of the mutant dynamics curve is approximately the same as the neutral equilibrium that was observed for neutral mutants (*N*_neut_). Hence, we hypothesized that the fixation probability of a disadvantageous mutant could be given by the Moran process formula assuming that the initial number of mutant viruses is given by *N*_neut_, that is by

(3) $$\frac{1-{\left(1/r\right)}^{N_{\mathrm{neut}}}}{1-1/r^{N_{\mathrm{viruses}}}}.$$

While this formula can predict the observed mutant fixation probability with reasonable accuracy for relatively low infection multiplicities (Fig. 3C, grey diamonds), the observed fixation probability is significantly larger than this measure at higher multiplicities. The reason for this discrepancy seems to be that in the context of our model formulation, there are two levels at which mutant and wild‐type viruses compete with each other: (i) Within a cell, a virus strain is picked for transmission with a probability given by the fraction of this strain in the cell. Hence, the mutant is neutral with respect to the wild‐type at this level. (ii) Between cells, the mutant is disadvantageous compared to the wild‐type because it has a reduced probability to enter a new target cell (given by *r* < 1). Therefore, the extent of the mutant fitness disadvantage is actually less than expressed by *r*, and the overall fitness of the mutant should be given by a value that lies between *r* and 1. The importance of this effect, however, should be influenced by the average multiplicity of the infected cells: If it is low, many cells contain either the mutant or the wild‐type virus alone, and then the within‐cell competition plays little role. In contrast, if the average infection multiplicity is high, most cells are likely to contain both mutant and wild‐type virus, and the within‐cell competition will play an important role. The overall fitness disadvantage of the mutant can thus be captured phenomenologically by an expression that places it between *r* and 1, weighed by the average infection multiplicity:

(4) $$r^{\prime }=1+\frac{\left(r-1)(m+1\right)}{2m}.$$

The parameter *m* denotes the average multiplicity among infected cells. If mutant fitness *r*’ is used in formula (3), we obtain a prediction that matches the fixation probability obtained in the computer simulation (red crosses superimposed on black circles in Fig. 3C).

The curve with black open circles in Figure 3A again depicts the results of simulations in which the mutant was placed randomly in one of the available cells, and where the fate of the mutant was tracked. Because the first mutant virus now arises in a cell that could also contain wild‐type viruses, the initial advantage of the mutant is less pronounced, as was the case for the neutral mutant. Hence, the mutant fixation probability is lower compared to that starting with a single mutant virus alone in a cell (closed black circles).

There is again a trade‐off between reduced fixation probabilities and the increased rates of mutant production with higher infection multiplicity (which is independent of mutant fitness). Again we recorded the time it takes for the mutant to fix for the first time. The trend is similar to that for neutral mutants: at moderate infection multiplicities, multiple infection speeds up mutant invasion, but at higher multiplicities, it slows down invasion (Figure 3D). The range of multiplicities over which mutant invasion is slower in the presence compared to the absence of multiple infection is wider for disadvantageous compared to neutral mutants (compare Figs. 2B and 3D). Additionally, the extent to which multiple infection slows down mutant invasion is significantly stronger for disadvantageous mutants. Hence, multiple infection is more detrimental for mutant invasion for disadvantageous compared to neutral mutants.

## Advantageous Mutants

**Figure 4:**
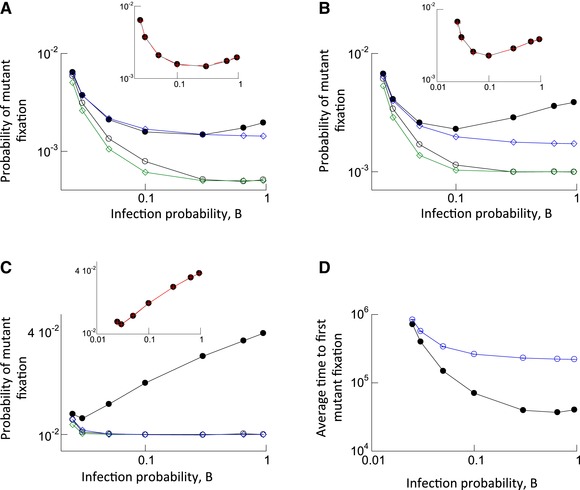
Evolutionary dynamics of advantageous mutants. (A) Fixation probability as a function of the infection probability, B. The blue line with diamonds shows the fixation probability in the absence of multiple infection, provided by formula (1). The green line shows the prediction of formula (2). The closed black circles show the fixation probabilities observed in the agent based simulation when one cell infected with one mutant virus is introduced into the system at equilibrium. The black open circles show the fixation probabilities observed in the computer simulation when one mutant virus is randomly placed into any of the available cells in system at equilibrium. The inset re‐plots the observed fixation probability shown in closed black circles, and the red crosses depict the prediction given by formula (3) when the composite fitness value *r*’ is calculated according to formula (4), as described in the text. Parameters were: B_1_ = 0.025, B_2_ = rB_1_, A = 0.02, L = 1, D = 0.01, *N* = 900, *r* = 1.0005. The number of simulation results for black closed circles was: 4,866,352; 5,371,603; 3,577,510; 4,091,305; 2,648,860; 1,691,486; and 1,272,341. For black open circles: 3,935,759; 4,490,230; 3,313,452; 4,309,990; 3,470,896; 4,837,538; and 5286726. (B and C) Same simulations, but with larger mutant advantages, *r* = 1.001 for (B) and *r* = 1.01 for (C). For (B), the number of simulation runs for the closed black circles are; 7,553,368; 6,920,249; 6,011,459; 5,067,733; 3,155,326; 1,939,997; and 1,507,107 . For black open circles: 17,417,910; 19,829,787; 14,557,581; 18,888,450; 15,088,002; 11,334,612; and 19,407,499. For (C), closed black circles: 7,519,349; 6,572,315; 5,445,510; 4,494,334; 2,831,424; 1,767,559; and 1,362,813. For (C), black open circles; 854,795; 773,698; 702,491; 681,172; 547,988; 407,116; and 333,571. Trends described in the text are statistically significant, according to the *Z* test for two population proportions. (D) Average time until 90% of the infected cell population contain the advantageous mutant for the first time (black closed circles), based on the agent‐based model with mutations and back‐mutations, as a function of the infection probability. Standard errors are plotted, but are hard to see. The numbers of simulation runs are: 19,839; 41,663; 82,828; 222,263; 316,597; 638,422; and 488,754. The blue line depicts the result of equivalent simulations in the absence of multiple infection. Again, standard errors are too small to see, and the number of simulation runs are: 130,825; 226,054; 282,790; 481,975; 494,045; 1,080,864; and 998,265. Parameters were: B_1_ = 0.025, B_2_ = rB_1_, A = 0.02, L = 1, D = 0.01, μ = 3 × 10^−5^, *N* = 900, *r* = 1.01. The trends described in the text are statistically significant, according to the two‐sample *t*‐test.

Finally, we examined the evolutionary dynamics of advantageous mutants, assuming different degrees of mutant advantages (0.05%, 0.1%, and 1%; Fig. 4A–C, respectively). The fitness advantage of the mutant was implemented similarly compared to the model for disadvantageous mutants: We assumed an overall infection probability that was 0.05%, 0.1%, and 1% higher than the value of the parameter B. When a mutant virus was selected to enter a target cell, this process was assumed to always succeed. When the wild‐type virus was selected, there was a 0.05%, 0.1%, and 1% probability of failure. In this way, the wild‐type virus had infection probability B, while the mutant virus had an overall higher infection probability. In the absence of multiple infection, the fixation probability is again given by formula (1) (see the blue lines in Fig. 4A–C) derived from the Moran process, which we verified numerically (not shown). The parameter *r* > 1 now measures the relative advantage of the mutant virus. As before, the green line shows formula (2), which is the Moran‐process prediction for the fixation probability assuming that virus population size is given by the total number of viruses across all cells (rather than the number of infected cells).

We again start from a single cell containing one mutant virus placed into a wild‐type virus population at equilibrium, and determine the mutant fixation probabilities (shown with black closed circles in Fig. 4A–C for different infection probabilities, and hence, multiplicities). The mutant fixation probability first declines with infection multiplicity (infection rate), and subsequently increases to levels that are larger than those observed without multiple infection. If the mutant has a larger advantage compared to the wild‐type, this increase in the fixation probability is more pronounced (compare panels A, B & C of Figure 4). Hence, for sufficiently advantageous mutants, multiple infection largely increases the chances of mutant fixation.

The insets in panels (A–C) of Figure 4 show that the fixation probability of the advantageous mutant is again accurately predicted by formula (3) derived from the Moran process, where the overall mutant fitness *r*’ is calculated by the empirical formula (4) (see red crosses superimposed on black circles). As before, this assumes that the initial number of mutant viruses is given by the neutral equilibrium (*N*_neut_, described in the context of neutral mutants above). This makes sense because the initial phase of mutant spread from the first cell (that only contains mutant virus) is similar for all mutant types as long as the fitness difference is not too large. Only after this initial virus dissemination does the competition between the two virus strains start to matter.

The open black circles show the results of simulations in which the mutant was placed randomly in any of the available cells once the wild‐type virus population had equilibrated. Now, a drastically different trend is observed: the observed mutant fixation probability declines monotonically with infection multiplicity, as is also the case in the curve predicted by formula (2) (Fig. 4A–C, compare black open circles and green line). The larger the extent of the mutant advantage, the closer the observed fixation probability is compared to the green line. In addition, we note that for more pronounced mutant advantages, the mutant fixation probability becomes largely independent of infection rate, and hence, multiplicity (Fig. 4C). This indicates that for advantageous mutants, the nature of the initial conditions plays a very important role in determining how multiple infection influences the probability of mutant fixation.

As before, we also considered the physiologically more relevant scenario where a wild‐type population at equilibrium is allowed to mutate with a probability *p*_mut_ per infection, thus, repeatedly giving rise to the mutant virus. We find that the time to mutant invasion (first mutant fixation) is always lower in the presence compared to the absence of multiple infection, and that an increase in multiplicity reduces the time until mutant invasion (Fig. 4D). This follows from the above observations that (i) the advantageous mutant fixation probability shows a weak dependence on multiplicity if the mutant is placed randomly into any of the cells, and (ii) the rate of mutant generation is faster for higher infection multiplicity. Hence, in the context of advantageous mutants, multiple infection speeds up mutant invasion.

## Altered Viral Yield from Multiply Infected Cells

While we assumed so far that the rates of virus production and cell death, and hence, total viral yield, were independent of multiplicity, the basic trends remain robust under the assumption that total viral yield either increases or decreases with infection multiplicity, as shown in Supporting Information Section 4.

## Discussion and Conclusion

We used computational models to investigate the spread dynamics of mutant viruses in the presence of multiple infection, assuming relatively simple settings where no viral complementation, inhibition, or recombination/reassortment occurred. Nevertheless, the dynamics were found to be complex. An interesting aspect concerns the early growth dynamics of neutral and disadvantageous virus mutants when rare. During the initial stages of the dynamics, the mutant population enjoys growth instead of drifting, similar to an advantageous mutant, because the number of mutant‐infected cells can grow by viral entry into uninfected as well as wild‐type‐infected cells, while the number of wild‐type infected cells can only increase thorough spread to uninfected target cells at this stage. Following this initial spread, the dynamics of these mutants become more typical, with neutral mutants undergoing neutral drift and disadvantageous mutants experiencing a selective disadvantage. In this phase, the probability of mutant viruses to spread is reduced at higher multiplicities. These patterns lead to the counter‐intuitive result that multiple infection can promote the presence of neutral or disadvantageous mutants in the short‐term, but reduces the chances to find those mutants in the longer term. This can complicate the interpretation of experimental data, as explored in the discussion of a specific experimental study about phage φ6 dynamics, presented in the Supporting Information Section 8. While a distinction between the short‐ and long‐term success of mutants does not apply to advantageous types, we again found that multiplicity reduced the long‐term invasion potential of mutants, although this trend was less pronounced, especially when the fitness advantage of the mutant was relatively large. Challenges associated with experimental tests of these kinds of dynamics are discussed in the Supporting Information Section 7.

If production of mutants by wild‐type virus is taken into account in the models, the effect of multiplicity on the rate at which mutants emerge and spread becomes more complex. While long‐term mutant invasion is predicted to be hampered by higher multiplicity, the rate at which new mutants are produced and successfully enter target cells is increased by multiple infection. The resolution of this trade‐off depends on the situation: For neutral and disadvantageous mutants, multiple infection can either speed up or slow the invasion of the mutant, depending on the average infection multiplicity in the system. For advantageous mutants, multiple infection is predicted to speed up mutant invasion. Therefore, multiplicity does not have a straightforward and consistent effect on the rate of mutant emergence and invasion. For example, the evolution of immune escape mutants in chronic infections that are controlled by ongoing immune responses is most likely accelerated by a higher infection multiplicity, since such mutants enjoy an instant fitness advantage. At the same time, however, other, equally important, evolutionary processes can be hampered at high multiplicities, such as the emergence of drug‐resistant mutants before treatment initiation (standing genetic variation). Such mutants typically have a certain selective disadvantage compared to drug‐sensitive viruses in the absence of treatment (Cong et al. 2007).

This work forms a foundation for further explorations of viral evolution that go beyond the basic dynamics considered here, including the consequences of recombination, reassortment, complementation, and inhibition between wild‐type and mutant viruses within the same cell, processes that likely shape evolutionary trajectories at high multiplicity of infection (MOI). For example, it has been shown that frequent coinfection can have an overall deleterious effect on the virus population due to the persistence of defective or inferior virus strains, which outweighs the advantages derived from viral sex at high multiplicities (Froissart et al. 2004). As another example, it has been shown that mutually beneficial interactions among genetically different virus strains that lead to increased viral output from the infected cell can promote coexistence, even in the presence of significant fitness differences (Leeks et al. 2018). Such interactions will be incorporated into our modeling framework in future studies.

Associate Editor: K. Lythgoe

## Supporting information


**Figure S1**. Schematic representation of the assumptions underlying the basic agent‐based computational modeling framework.
**Figure S2**. Simulations of wild‐type dynamics in the agent‐based model.
**Figure S3**. The average infection multiplicity is varied by changing the infection probability of the virus, B, as shown. The average multiplicity was determined by running the simulation repeatedly (10,000 runs), and taking the average value at a specific time point during the equilibrium phase of the dynamics.
**Figure S4**. Fixation probability of a neutral mutant in the agent based model where the rate of virus production is a saturating function of infection multiplicity.
**Figure S5**. Mutant fixation probability as a function of the infection probability, B, assuming increased viral output in multiply infected cells.
**Figure S6**. Same plot as Figure S4 in main text, but assuming that both the rate of virus production and the death rate of infected cells increase equally with infection multiplicity.
**Figure S7**. Mutant fixation probability as a function of the infection probability, B, assuming that viral output from infected cells declines with multiplicity.
**Figure S8**. Time to mutant fixation in a model without back‐mutation.
**Figure S9**. Mutant fixation probability as a function of the infection probability, B.
**Figure S10**. Average mutant dynamics in the presence (red) and absence (blue) of multiple infection, based on repeated realizations (100,000) of the agent‐based model without mutational processes.
