# Supplementary material for: Multiple infection of cells changes the dynamics of basic viral evolutionary processes
Source: Evol Lett. 2018 Dec 31;3(1):104–15. doi: 10.1002/evl3.95 (PMC6369963; doi:10.1002/evl3.95)
Supplement: Supplementary file 1 — Figure S1. Schematic representation of the assumptions underlying the basic agent‐based computational modeling framework. Figure S2. Simulations of wild‐type dynamics in the agent‐based model. Figure S3. The average infection multiplicity is varied by changing the infection probability of the virus, B, as shown. The average multiplicity was determined by running the simulation repeatedly (10,000 runs), and taking the average value at a specific time point during the equilibrium phase of the dynamics. Figure S4. Fixation probability of a neutral mutant in the agent based model where the rate of virus production is a saturating function of infection multiplicity. Figure S5. Mutant fixation probability as a function of the infection probability, B, assuming increased viral output in multiply infected cells. Figure S6. Same plot as Figure S4 in main text, but assuming that both the rate of virus production and the death rate of infected cells increase equally with infection multiplicity. Figure S7. Mutant fixation probability as a function of the infection probability, B, assuming that viral output from infected cells declines with multiplicity. Figure S8. Time to mutant fixation in a model without back‐mutation. Figure S9. Mutant fixation probability as a function of the infection probability, B. Figure S10. Average mutant dynamics in the presence (red) and absence (blue) of multiple infection, based on repeated realizations (100,000) of the agent‐based model without mutational processes. [file EVL3-3-104-s001.pdf]

Supplementary Materials:
Additional information and analysis for paper: “Multiple infection of cells changes the dynamics of basic viral evolutionary processes”

Dominik Wodarz[1,3*], David N. Levy[2], and Natalia L. Komarova[3,1]

1: Department of Ecology and Evolutionary Biology, Steinhaus Hall, University of Cali fornia, Irvine CA 92697

2: Department of Basic Science, 921 Schwartz Building, New York University College of Dentistry, New York, NY 10010, USA.
3: Department of Mathematics, Rowland Hall, University of California, Irvine, CA 92697.

Table of Contents:

1. The agent-based modeling framework…………………………………………...2
2. ODE descriptions of the agent-based model……………………………………..…4
3. Varying the average infection multiplicity in the agent-based model……………..…6
4. Viral yield and infection multiplicity………………………………………………..7
5. Time to neutral mutant invasion: simulations without back mutations…………13
6. Limited infection multiplicity…………………………………………………………15
7. Challenges associated with experimental tests of theoretical predictions……..16
8. Theory and existing data……………………………………………………………17

1. The agent-based modeling framework.

The basic agent-based computational model is described in words in the main text. To complement this description, we provide a schematic representation of the basic assumptions underlying the model in Figure S1.

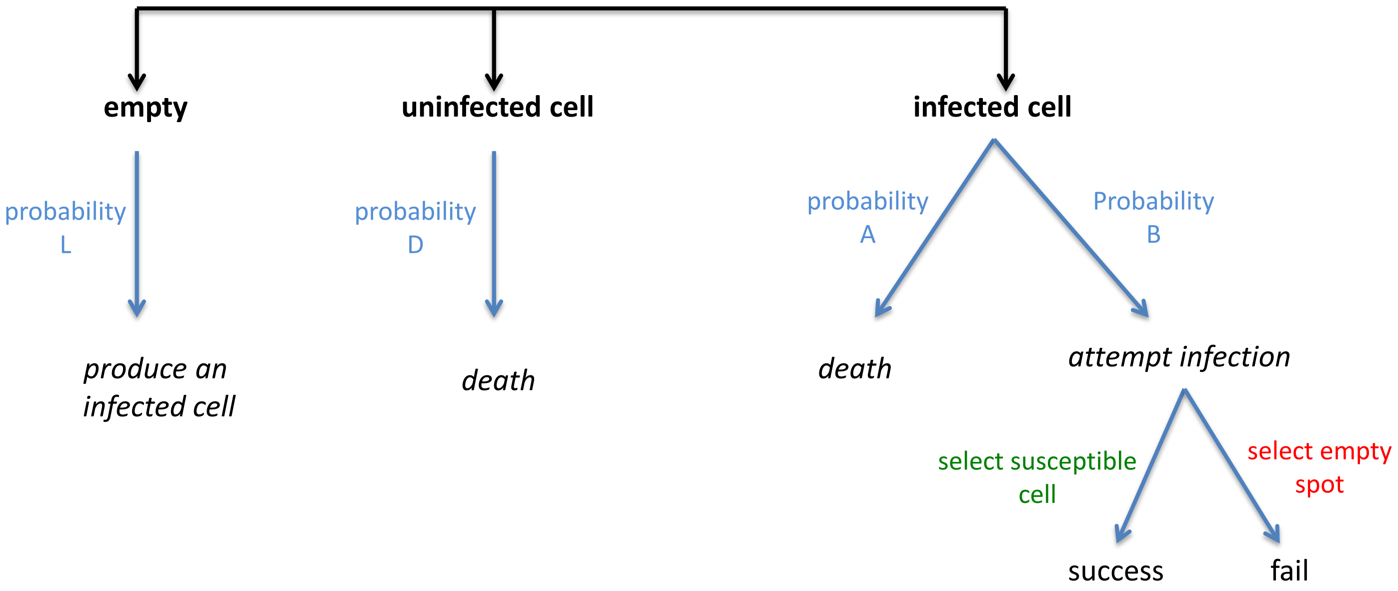

Figure S1: *Schematic representation of the assumptions underlying the basic agent-based computational modeling framework. For description, see main text.*

Additional notes about mutant generation in the agent-based model: In the agent-based model, mutation is coupled to the virus replication / infection process. During an infection event, an offspring virus particle is picked for transfer to a new target cell. Let us assume that all viruses in the infected cell are wild-type. With a certain probability, the chosen offspring virus can be mutated, and the mutated virus is then placed into a randomly chosen target cell. When running simulations to determine the mutant fixation

probability, we explored a scenario where one mutant was initially placed randomly into any cell in the system (uninfected or already infected), to imitate a setting where the mutant virus was just generated by mutation from wild-type. We can think of two different categories of viral life cycle, and this formulation should apply to both, which is explained as follows.

(i) An example of the first scenario is the retrovirus HIV. Most mutations occur upon reverse transcription, which happens at the very beginning of the life-cycle, following infection. Hence, the mutation is generated during the infection process. In terms of our model algorithm, an offspring virus (wild-type) in the original infected cell is transferred to a new target cell. With a probability (1-μ), the virus remains wild-type. With a probability μ, a mutation occurs and the virus is turned into a mutant in the target cell. If the target cell is uninfected, then the mutant will be the only virus in the newly infected cell. If the target cell is already infected, the mutant virus will be added to that cell. Therefore, it makes sense that a mutant can appear in any of the cells, either uninfected or infected.

(ii) Let us now consider a different virus life-cycle, where the mutant is produced upon virus replication in the original infected cell. In our algorithm, we pick a single offspring virus particle for infection. Now, with a probability (1-μ), this will have remained a wild-type virus during replication. With a probability μ, a mutation event occurred during replication and the offspring virus particle under consideration will be a mutant. The offspring virus (mutant or wild-type) is transferred to a randomly chosen target cell, which might be uninfected or already infected. Hence, the same algorithm applies.

Again, it makes sense to assume that the mutant can appear in any of the cells, including uninfected cells.

2. ODE descriptions of the agent-based model

The average dynamics of the stochastic agent-based model can be captured by ordinary differential equations. Denoting uninfected cells by y_0 and cells infected by i viruses by y_i, the equations are given as follows:

$$\frac{dy_0}{dt} = \lambda(1 - \frac{x+v}{k}) - dy_0 - \frac{\beta y_0 v}{k},$$

$$\frac{dy_i}{dt} = \frac{\beta y_{i-1} v}{k} - ay_i - \frac{\beta y_i v}{k}, \qquad i > 0, \qquad (1)$$

$$\text{where} \quad v = \sum_{i=1}^{\infty} y_i.$$

The variable v denotes the sum of all infected cells, which is proportional to the number of free viruses if free virus is in a quasi-steady state (Nowak and May, 2000). Uninfected target cells are produced with a rate λ, and this process is density-dependent, as in the agent-based model. These cells die with a rate d and become infected with a rate β. Infected cells die with a rate a, and can be further infected with a rate β. For numerical integration, this ODE formulation requires truncation at a maximum multiplicity, n, which needs to be large enough in computer simulations such that the population y_n remains negligible (Phan and Wodarz, 2015). The virus establishes a persistent infection if its

basic reproductive ratio, $R_0 = \frac{\lambda\beta}{(\lambda + kd)a}$, is greater than one. In this case, the dynamics converge to a stable equilibrium given by

$$y_0^* = \frac{ak}{\beta}, \qquad \sum_{i=1}^{n} y_i^* = \frac{k(\lambda\beta - \lambda a - dak)}{\beta(\lambda + ak)}.$$

In the agent-based model, the populations will fluctuate around this equilibrium, due to the stochastic nature of the system, and the population of cells will be characterized by a given average infection multiplicity (Figure S2 A & B).

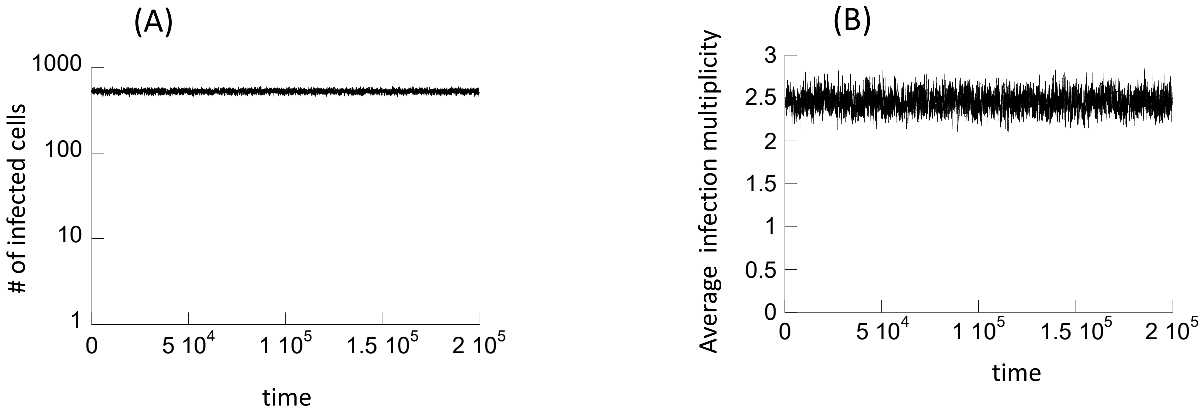

Figure S2: Simulations of wild-type dynamics in the agent-based model. *(A) Over time, the number of infected cells converges towards and equilibrium value, around which the population fluctuates stochastically. A single typical run of the simulation is shown. (B) The average infection multiplicity across all infected cells also fluctuates around a steady state. Again, single typical simulation run is shown. B=0.025, A=0.02, L=1, D=0.01, N=900.*

Next, we consider both a mutant and a wild-type virus. Denoting uninfected cells by y_{00} and cells infected with i copies of the wild-type virus and j copies of the mutant virus by y_{ij}, the equations are given as follows:

$$\frac{dy_{00}}{dt}=\lambda(1-\frac{y_{00}v_1+v_2}{k})-dy_{00}-\frac{\beta_1 y_{00}v_1}{k}-\frac{\beta_2 y_{00}v_2}{k},$$

$$\frac{dy_{ij}}{dt}=\frac{\beta_1 y_{i-1,j}v_1}{k}+\frac{\beta_2 y_{i,j-1}v_2}{k}-ay_{i,j}-\frac{\beta_1 y_{i,j}v_1}{k}-\frac{\beta_2 y_{i,j}v_2}{k}, \qquad i+j>0, \qquad (2)$$

where $v_1=\sum_{i+j>0}\frac{i}{i+j}y_{ij},\ v_2=\sum_{i+j>0}\frac{j}{i+j}y_{ij}.$

The variables v_1 and v_2 represent the sum of the fractions of the respective virus strains in the cell. This is proportional to the free virus populations if the rate of virus production is independent of multiplicity and if the virus is assumed to be in a quasi-steady state. The relative fitness of the two virus strains is determined by differences in the infection rates, β_1 and β_2. If these two rates are identical, the two virus strains are competitively neutral. For numerical integration, the system is truncated by only retaining the equations with $i+j\leq n$, where n is sufficiently large.

3. Varying the average infection multiplicity in the agent-based model

One of the parameters that determines the average infection multiplicity in the agen-based model is the infection probability, B. Therefore, to explore dependence of evolutionary outcomes on multiplicity, we varied the parameter B, and the relationship between the value of B and the average multiplicity under the parameters used in the figures is shown in Figure S3.

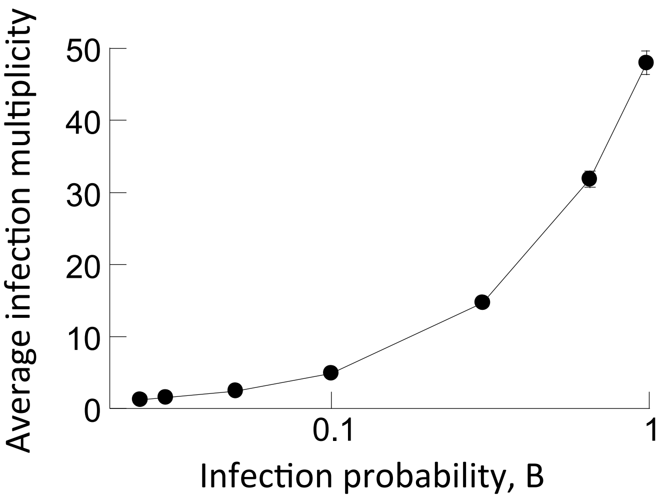

Figure S3: *The average infection multiplicity is varied by changing the infection probability of the virus, B, as shown. The average multiplicity was determined by running the simulation repeatedly (10,000 runs), and taking the average value at a specific time point during the equilibrium phase of the dynamics. Standard deviations are plotted (almost not visible due to relatively small value). Base parameters are given as follows. B=0.025, A=0.02, L=1, D=0.01, N=900.*

4. Viral yield and infection multiplicity

The analysis performed in the main text assumed that the amount of virus produced by infected cells during their life-spans (viral yield) is the same regardless of the infection multiplicity. This means that cellular factors limit the rate of virus production, and introduces an element of intracellular competition among the different virus strains. This section explores the effect of relaxing this assumption.

We can assume that the viral output from infected cells goes up with infection multiplicity trough an increase in the rate of virus replication in multiply infected cells (Dormond et al., 2009; Ferreira et al., 2005; Gueret et al., 2002; Perez−Cruet et al.,

1994; Rothmann et al., 1998). In particular, cells containing two viruses would produce twice as many offspring viruses, cells infected with three viruses would produce three times the amount of offspring virus, etc. This, however, would give rise to a positive feedback loop where higher multiplicity increases the rate of viral replication, which in turn increases the infection multiplicity. The biologically most reasonable assumption in this context would be that the rate of virus production is a saturating function of the number of viruses that are present in the cell. Hence, in the model, the probability for an infected cell to pass on the virus to a target cell is not given by B anymore, but by $B(V)(1+\varepsilon)/(V+\varepsilon)$, where V denotes the total number of viruses in a cell. The larger the constant ε, the more the rate of virus production (and hence total yield) increases with multiplicity before converging to an asymptote. Thus, $\varepsilon=0$ corresponds to the case where viral output is independent of the multiplicity of infection, and $\varepsilon \rightarrow \infty$ corresponds to the output increasing in an unlimited fashion with multiplicity. We investigated the fixation probability in the context of a neutral mutant. As the initial condition, we placed a single cell with one mutant virus into a wild-type population at equilibrium and recorded the mutant fixation probability as a function of the saturation constant ε. The results are shown in Figure S4 (black filled circles). For low values of ε, the fixation probability is close to the one observed for neutral mutants where virus output was assumed independent of infection multiplicity. As the value of ε increases (more pronounced increase in viral replication/output in multiply infected cells), the mutant fixation probability declines. This makes intuitive sense, because the average infection multiplicity in the cells rises with increasing values of ε. The green line (Figure S4) shows the reference value $1/N_{viruses}$, i.e. the expectation under neutral evolutionary

theory. As before, the observed mutant fixation probability is significantly higher than the one predicted by neutral evolutionary theory (green line), for the same reason as given in the simpler versions of the model in the main text, where the rate of virus production was independent of multiplicity: the mutant dynamics first display a spread phase before the number of mutant viruses converges to a neutrally stable equilibrium (N_{neut}). As in the simpler model in the main text, the fixation probability is again given by $N_{neut}/N_{viruses}$, as shown by the red crosses that are superimposed on the black circles in Figure S4.

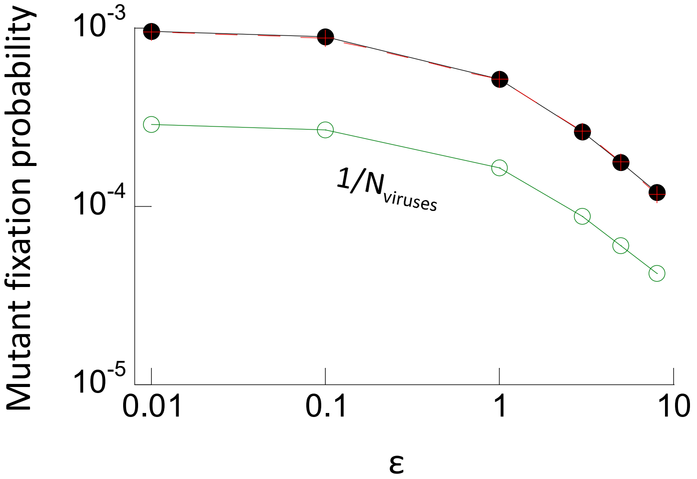

Figure S4: *Fixation probability of a neutral mutant in the agent based model where the rate of virus production is a saturating function of infection multiplicity. The fixation probability is shown as a function of the parameter ε, which determines how quickly saturation occurs. Higher values of ε correspond to a more pronounced increase in viral output with multiplicity. The green line again depicts the value of* $1/N_{viruses}$*. The red line plots the value of* $N_{neut}/N_{viruses}$*, which successfully predicts the observed fixation probabilities. Parameters were: B=0.025, A=0.02, L=1, D=0.01, N=900. The number of simulation runs for increasing values of ϵ were: 119736073, 117908559, 104741112, 87608798, 75812069, 64365150. The trends described in the text are statistically significant, according to the Z test for two population proportions (very low p values, not shown).*

We also demonstrate the effect of increasing the parameter ε on the fixation probabilities by re-plotting Figure 2A in the main text for different value of ε (Figure S5).

The same dependence of the neutral mutant fixation probability on the infection rate (multiplicity) is observed, with higher values of ε shifting the curve towards lower fixation probabilities. Therefore, the overall trends described in the main text are not tied to the assumption that the rate of virus production is independent of multiplicity.

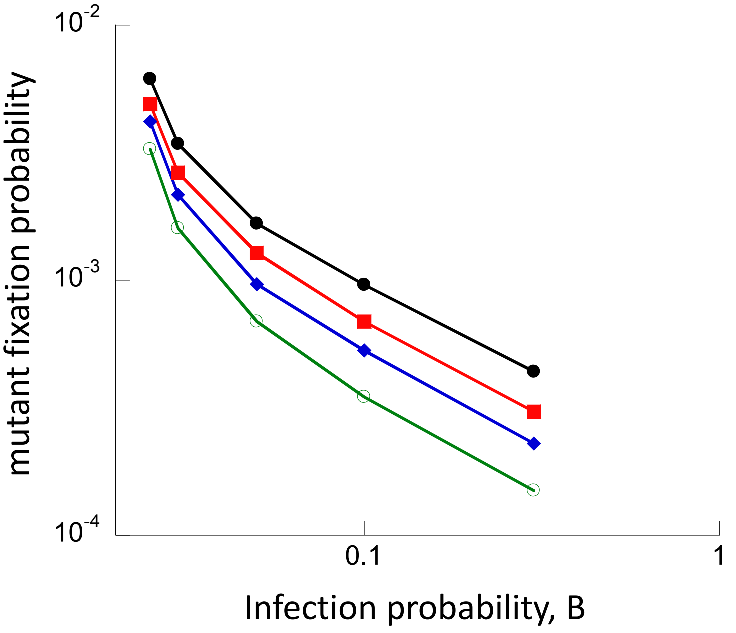

Figure S5: *Mutant fixation probability as a function of the infection probability, B, assuming increased viral output in multiply infected cells. The black line with closed circles is the same line as the one presented in Figure 2A in the main text, assuming that viral output from infected cells is independent of multiplicity. The colored lines are equivalent simulations, but assuming that viral output from infected cells increases with multiplicity to varying degrees, captured by the parameter ϵ. ϵ=0.5, 1, 2 for the red, blue, and green lines, respectively. All other parameters and conditions are the same as specified for Figure 2A.*

In these simulations, we assumed that the total virus yield per cell increases with multiplicity, due to faster virus replication in multiply infected cells. If an increase in virus reproduction in multiply infected cells, however, is matched by an identical increase in

the death rate of infected cells, the total virus yield per cell becomes independent of multiplicity. To explore this, we assumed that the viral replication rate is a function of multiplicity as before, given by B*V(1+ε)/(V+ ε), and that the rate of infected cell death has the same dependency, given by A*V(1+ε)/(V+ ε). Figure S6 plots the neutral mutant fixation probability as a function of the extent to which the replication and death parameters increase with multiplicity, ε. As expected, no change is observed.

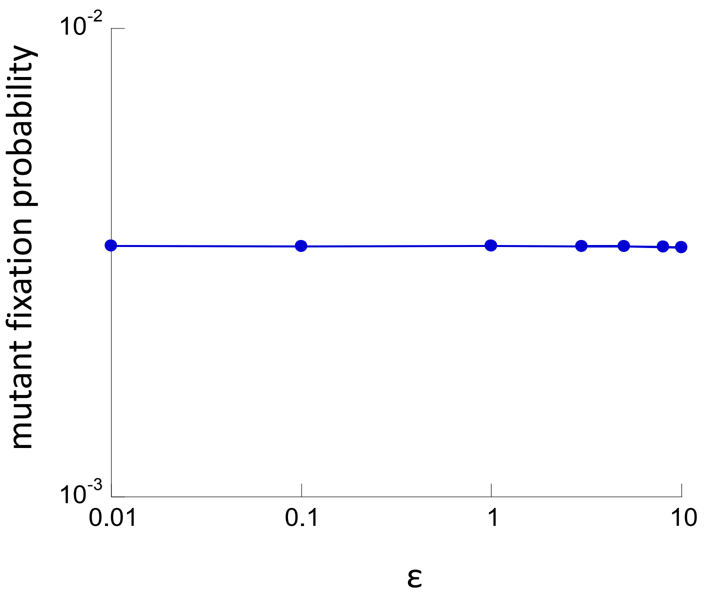

Figure S6: *Same plot as Figure S4 in main text, but assuming that both the rate of virus production and the death rate of infected cells increase equally with infection multiplicity. The amount of increase is given by the parameter ϵ.*

Finally, it is also possible that multiple infection results in less overall virus production (lower yield) compared to single infection. This might be due to over-exploitation of cellular resources. It can occur if the death rate of infected cells increases with multiplicity to a larger extent than the viral replication rate. This was captured as follows. In addition to the viral replication rate increasing asymptotically with multiplicity,

we assume that that death probability of infected cells increases according to the same function, but to a larger extent: $B(V)(1+\varepsilon_2)/(V+\varepsilon_2)$. We set $\varepsilon_2 > \varepsilon$, such that an increase in multiplicity results in a stronger increase in the infected cell death probability than in the viral replication rate. The total viral yield produced during the life-span of an infected cell now declines with multiplicity. Figure S7 plots the fixation probability of neutral mutants against the infection probability, for different values of ε_2.

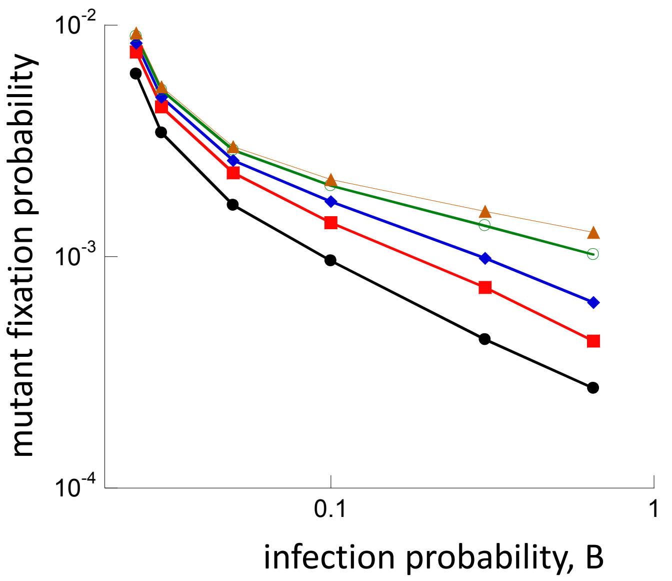

Figure S7: *Mutant fixation probability as a function of the infection probability, B, assuming that viral output from infected cells declines with multiplicity. The black line with closed circles is the same line as the one presented in Figure 2A in the main text, assuming that viral output from infected cells is independent of multiplicity. The colored lines are equivalent simulations, but assuming that both the viral production rate and the death rate of infected cells increases with multiplicity, with the increase being more pronounced for the death rate, expressed by ε2> ε. ϵ2=1, 2.5, 10, 100 for the red, blue, green, and brown lines, respectively. All other parameters are the same as specified for Figure 2A.*

This was done for simulations where a single cell containing mutant virus only was placed into the wild-type population at equilibrium. For reference, the black line replots the simulation for the assumption that virus parameters are independent of multiplicity

(also shown in Figure 2A, main text). The colored lines plot the same kind of graph for different values of ε_2. The same general relationship is observed (Figure S7). The more virus yield declines with multiplicity (higher values of ε_2), the higher the fixation probability of the neutral mutant (Figure S7). The reason is that a decline of overall virus yield with higher multiplicities results in reduced levels of multiple infection, which promotes the chances for the mutant to invade.

5. Time to neutral mutant invasion: simulations without back mutations

The main text considers a version of the model that includes mutational processes. This model was used to investigate the relationship between infection multiplicity and the time to mutant invasion, starting from a wild-type only population at equilibrium. The model included both forward and back-mutations. Having both forward and back-mutations makes biological sense, because we consider point mutations in individual base pairs, and the rates of forward and back-mutations are identical in this case. Figure 3B in the main text plots the time until the mutant reaches 90% of the total virus population. Here, we consider a version of the model that does not include back-mutations, for the purpose of comparison. In the absence of back-mutations, we consider time to fixation rather than time until the mutant makes up 90% of the total population. This is because uni-drectional mutations ensure that the wild-type virus is not re-created from the mutant. Results are shown in Figure S8.

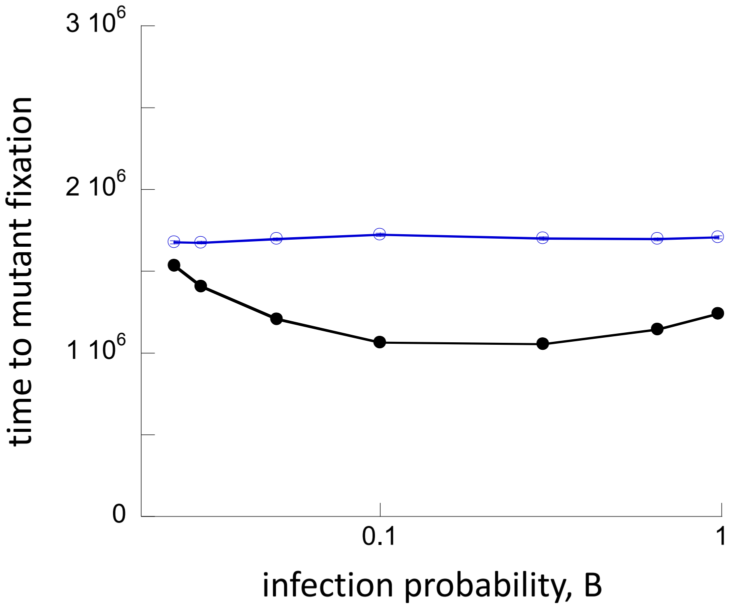

Figure S8: *Time to mutant fixation in a model without back-mutation. Blue depicts results in the absence of multiple infection, black in the presence of multiple infection. Parameters and conditions are identical to Figure 3B in the main text.*

Similar to the back-mutation model, the time to invasion without back-mutations first declines with infection multiplicity and subsequently increases (compare Figure 3B and S8). This subsequent increase in time to invasion, however, is much less pronounced in the absence of back-mutations because the mutant essentially has an advantage (due to the unidirectional production of mutants from wild type).

6. Limited infection multiplicity

In the models explored in the main text, the amount of multiple infection is in principle not limited. It is determined by the replication rate of the virus and the death rate of the infected cells. In several virus infections, however, it has been reported that the virus can limit the amount of multiple infection. For example in HIV-1 infection, the virus induces the down-modulation of the CD4 T cell receptor some time after viral entry into the cell (Levy et al., 2004), after which no further superinfections can occur. In phages, it has been reported that maximally 2-3 viruses might be simultaneously present in a cell (Turner et al., 1999), although the mechanism underlying this observation requires further investigation. To determine the effect of artificially limiting the infection multiplicity, we determined the fixation probability of neutral mutants as a function of the infection rate under the assumption that a cell can contain maximally two viruses. After this multiplicity has been achieved, no further superinfection was allowed. We concentrate on the scenario where a single cell containing only mutant virus is placed into a wild-type virus population at equilibrium. The results are shown in Figure S9. The black line is the same as the one in Figure 2A in the main text. The red line represents the results of the limited multiplicity scenario. For low infection rates, multiplicity is naturally limited to low values due to the slow infection kinetics. Hence the results of the two scenarios are very similar. For higher infection rates, average multiplicities go above two in the unlimited scenario, so the results of the limited and unlimited multiplicity simulations diverge. Limited infection multiplicity leads to higher fixation probabilities of the neutral mutant, because the extent of intracellular competition is

reduced. The general dependence of the fixation probability on multiplicity, however, remains qualitatively the same.

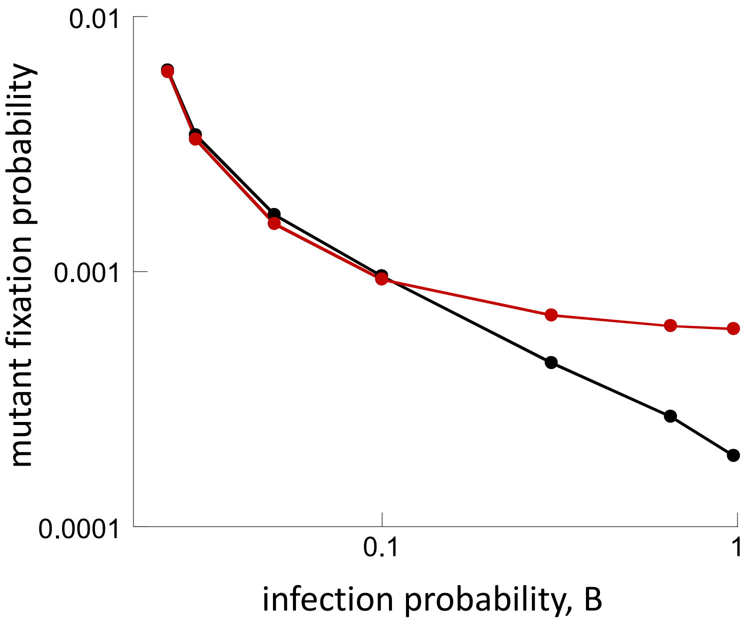

Figure S9: *Mutant fixation probability as a function of the infection probability, B. The black line with closed circles is the same as in Figure 2A, and the red line re-plots equivalent results under the assumption that maximally 2 viruses can be simultaneously present in a cell.*

7. Challenges associated with experimental tests of theoretical predictions.

To test some of the model predictions presented in this paper, an in vitro system would be required where a virus population is maintained at steady state with its target cell population, into which a mutant virus is introduced and the mutant spread is documented. Further, the ability of the virus to productively super-infect cells would have to be experimentally manipulated. We are not aware that a feasible in vitro virus-

host cell system currently exists that would allow such an experimental test. With such a system, it would be possible to experimentally investigate the early growth dynamics of a mutant virus in the presence and absence of multiple infection. Predictions regarding fixation probabilities are even less tractable to test because of the very large number of experimental repeats required for an accurate assessment.

8. Theory and existing data

An important aspect of theoretical work is to use the results to interpret experimental data. While the dynamics of mutant fixation have not been studied in settings that vary the infection multiplicity, the number of mutants has been quantified in experiments where the bacteriophage φ6 was passaged under low and high infection multiplicity scenarios (Dennehy et al., 2013). It was found that after 300 generations, genetic diversity was larger at low compared to high infection multiplicities, and that this difference was mostly due to the presence of mutations in non-coding regions of the genome. These were considered likely to be neutral mutations (Dennehy et al., 2013), although mutations in non-coding regions need not necessarily be neutral (Cuevas et al., 2012; Peris et al., 2010). These results suggested that processes occurring at high infection multiplicity (e.g. reassortment of genomic segments, sexual exchange), did not contribute to viral genetic diversity (Dennehy et al., 2013).

The models analyzed in our study made predictions about the average dynamics of neutral mutant viruses over time, which might be useful for the interpretation of these

experimental data. In the presence of multiple infection, the dynamics were characterized by two phases: (i) An early growth phase was observed, where the dynamics resemble those of an advantageous mutant, which is not seen with neutral mutants in the absence of multiple infection. Hence, we expect that multiple infection promotes the spread of neutral mutants during this initial phase, which is counter to the experimental observations (Dennehy et al., 2013). (ii) This initial phase is followed by convergence of the dynamics to a neutral equilibrium, the level of which predicts the long-term fixation / extinction probability of the mutant. Fixation is less likely with than without multiple infection, and declines with higher infection multiplicities. Stated in another way, the mutant virus is more likely to go extinct in the presence compared to the absence of multiple infection, and higher multiplicities further promote mutant extinction. Therefore, during this longer-term phase, the number of neutral viruses is predicted to be larger at low compared to high multiplicities. This is in agreement with the experimental data on the evolution of phage φ6 (Dennehy et al., 2013), indicating that the timing of the observation could play an important role for the results.

Another complication in the interpretation of the experimental data concerns the experimental measure under consideration. The model suggests that different results can be obtained about the average number of mutants at low and high infection multiplicities depending on whether the number of mutant-infected cells are counted, or whether the amount of free virus is compared. This is demonstrated with computer simulations in Figure S10.

As initial conditions, a model simulation with wild-type virus only was allowed to equilibrate, and 10% of the infected cell population was sampled to start a new growth phase. A small number of mutant viruses was added to this pool of cells and the resulting growth curves were recorded. This might mimic a virus passage, which was part of the experiments performed by Dennehy et al (Dennehy et al., 2013). Many repeats of such runs were performed, and the average population sizes, as well as standard errors are plotted over time in Figure S10.

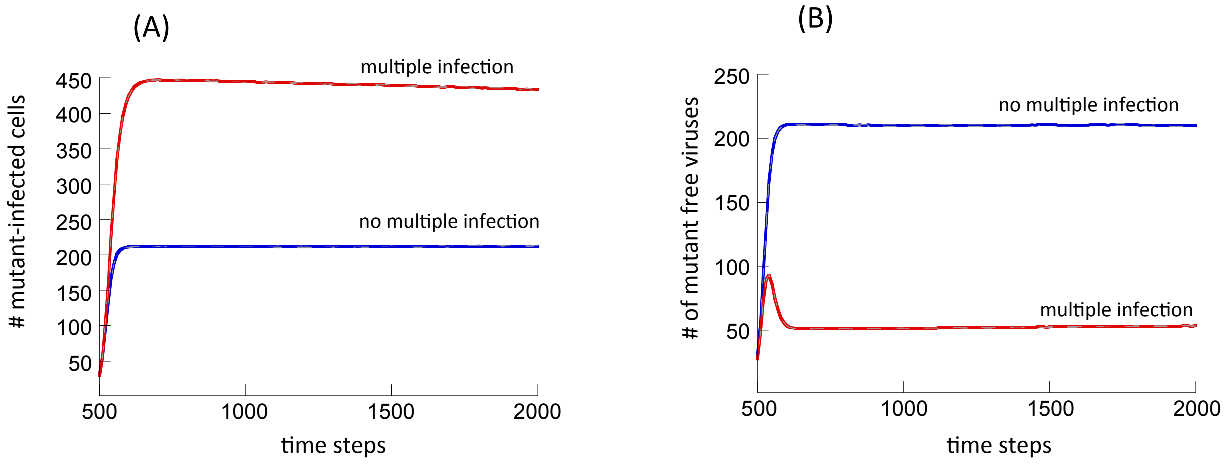

Figure S10: *Average mutant dynamics in the presence (red) and absence (blue) of multiple infection, based on repeated realizations (100,000) of the agent-based model without mutational processes. Grey dashed lines depict the standard errors (very small, hard to see). The simulations were started with wild-type virus only, until the system equilibrated. Then, 10% of the wild-type-infected cells were randomly selected, and renewed growth was simulated, together with a minority population of mutants (30% of the wild-type population). This might mimic the basic virus passage procedures in phage experiments reported by Dennehy et al (Dennehy et al., 2013). (A) The number of mutant-infected cells is plotted. (B) The sum of the mutant fractions across all infected cells is plotted, which is proportional to the amount of free virus. Parameters were: B=0.025, A=0.02, L=1, D=0.01, N=900.*

Figure S10A shows that if the number of mutant-infected cells is compared, the number is larger in the presence compared to the absence of multiple infection. In contrast,

Figure S10B shows the opposite if a measure proportional to the free virus population is compared. Even though more mutant-infected cells are predicted in the presence of multiple infection, if the mutant virus is significantly diluted by wild-type copies within those cells, fewer mutant free viruses will be observed in the presence of multiple infection. The reason is the assumption that the rate of mutant virus production is proportional to the fraction of the mutant in the cell.

We note that these simulations do not aim to reproduce the experiments by Denehy et al. (Dennehy et al., 2013), but to highlight complexities resulting from the multiple infection dynamics investigated here that could impact the interpretation of such data. There are uncertainties about processes in the experiments which make it challenging to construct models and simulations that exactly match the experimental conditions. For example it is unclear when the mutants are generated. A mix of mutants might be present in the initial inoculum, and their relative abundances might change during the growth and passage phases as a result of differential fitness. Alternatively, or in addition, mutants might be generated during the viral growth phases and invade. Irrespective of these details, however, the challenges to the interpretation of such experimental data, as outlined above, should apply and are important to keep in mind.

In summary, the models have identified two factors that can impact whether mutant spread is intensified in the presence or absence of multiple infection. The timing after mutant introduction can determine the result, and so can the particular measure of

mutant spread. These complexities are important to keep in mind when interpreting experimental data.

References

Cuevas, J. M., Domingo-Calap, P., Sanjuan, R., 2012. The fitness effects of synonymous mutations in DNA and RNA viruses. Mol Biol Evol 29, 17-20, doi:10.1093/molbev/msr179.

Dennehy, J. J., Duffy, S., O'Keefe, K. J., Edwards, S. V., Turner, P. E., 2013. Frequent coinfection reduces RNA virus population genetic diversity. J Hered 104, 704-12, doi:10.1093/jhered/est038.

Dormond, E., Perrier, M., Kamen, A., 2009. From the first to the third generation adenoviral vector: what parameters are governing the production yield? Biotechnology advances 27, 133-144.

Ferreira, T. B., Alves, P. M., Gonçalves, D., Carrondo, M., 2005. Effect of MOI and medium composition on adenovirus infection kinetics. Animal cell technology meets genomics. Springer, pp. 329-332.

Gueret, V., Negrete-Virgen, J. A., Lyddiatt, A., Al-Rubeai, M., 2002. Rapid titration of adenoviral infectivity by flow cytometry in batch culture of infected HEK293 cells. Cytotechnology 38, 87-97.

Levy, D. N., Aldrovandi, G. M., Kutsch, O., Shaw, G. M., 2004. Dynamics of HIV-1 recombination in its natural target cells. Proc Natl Acad Sci U S A 101, 4204-9.

Nowak, M. A., May, R. M., 2000. Virus dynamics. Mathematical principles of immunology and virology. Oxford University Press.

Perez-Cruet, M., Trask, T., Chen, S. H., Goodman, J., Woo, S., Grossman, R., Shine, H., 1994. Adenovirus-mediated gene therapy of experimental gliomas. Journal of neuroscience research 39, 506-511.

Peris, J. B., Davis, P., Cuevas, J. M., Nebot, M. R., Sanjuan, R., 2010. Distribution of fitness effects caused by single-nucleotide substitutions in bacteriophage f1. Genetics 185, 603-9, doi:10.1534/genetics.110.115162.

Phan, D., Wodarz, D., 2015. Modeling multiple infection of cells by viruses: Challenges and insights. Math Biosci 264, 21-8, doi:10.1016/j.mbs.2015.03.001.

Rothmann, T., Hengstermann, A., Whitaker, N. J., Scheffner, M., zur Hausen, H., 1998. Replication of ONYX-015, a potential anticancer adenovirus, is independent of p53 status in tumor cells. Journal of virology 72, 9470-9478.

Turner, P. E., Burch, C. L., Hanley, K. A., Chao, L., 1999. Hybrid frequencies confirm limit to coinfection in the RNA bacteriophage phi6. J Virol 73, 2420-4.
